# The TatD-like DNase of *Plasmodium* is a virulence factor and a potential malaria vaccine candidate

**DOI:** 10.1038/ncomms11537

**Published:** 2016-05-06

**Authors:** Zhiguang Chang, Ning Jiang, Yuanyuan Zhang, Huijun Lu, Jigang Yin, Mats Wahlgren, Xunjia Cheng, Yaming Cao, Qijun Chen

**Affiliations:** 1 Key Laboratory of Zoonosis, Jilin University, Xi An Da Lu 5333, Changchun 130062, China; 2 Institute of Microbiology, Tumour and Cellular Biology, Karolinska Institutet, Nobels väg 16, S-171 77 Stockholm, Sweden; 3 Department of Pathogen Biology, Fudan University, Handan Road 220, Shanghai 200433, China; 4 Department of Immunology, China Medical University, Puhe Road 77, Shenyang 110122, China; 5 Key Laboratory of Zoonosis, Shenyang Agricultural University, Dongling Road 120, Shenyang 10866, China

## Abstract

Neutrophil extracellular traps (NETs), composed primarily of DNA and proteases, are released from activated neutrophils and contribute to the innate immune response by capturing pathogens. *Plasmodium falciparum*, the causative agent of severe malaria, thrives in its host by counteracting immune elimination. Here, we report the discovery of a novel virulence factor of *P. falciparum*, a TatD-like DNase (PfTatD) that is expressed primarily in the asexual blood stage and is likely utilized by the parasite to counteract NETs. PfTatD exhibits typical deoxyribonuclease activity, and its expression is higher in virulent parasites than in avirulent parasites. A *P. berghei* TatD-knockout parasite displays reduced pathogenicity in mice. Mice immunized with recombinant TatD exhibit increased immunity against lethal challenge. Our results suggest that the TatD-like DNase is an essential factor for the survival of malarial parasites in the host and is a potential malaria vaccine candidate.

## Abstract (web summary)

Neutrophil extracellular traps (NETs), composed of DNA and proteases, contribute to the innate immune response by capturing pathogens. Here, Chang *et al*. identify a NET-degrading DNase in *Plasmodium* parasites and show that immunization with this protein provides protective immunity in a malaria mouse model.

## Graphical abstract

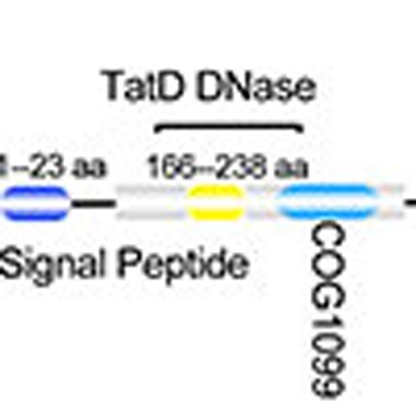

On activation by initial microbial contact, neutrophils and macrophages export filamentous elements composed of DNA and proteases, which confine the invading pathogens^1^,^2^. These filamentous structures are known as neutrophil extracellular traps (NETs) and have been demonstrated to contribute to pathogen capture as part of the innate immune response^3^,^4^. Correspondingly, some pathogens successfully escape the trap by secreting DNases to degrade the DNA^5^,^6^. The capacity of the released DNases to counteract NET confinement renders them microbial virulence factors^7^,^8^,^9^,^10^. Furthermore, plasma haem can induce the formation of NETs *in vitro*^11^, and haem is abundantly released in the host-blood circulation on the rupture of *Plasmodium falciparum*-infected erythrocytes^12^,^13^. NET structures were observed in the skin-associated peripheral blood of a *P. falciparum*-infected child with uncomplicated malaria^14^. *P. falciparum*, the causative agent of severe malaria, thrives in its host by counteracting immune elimination^15^,^16^,^17^.

We hypothesize that NETs may be an important innate immune factor for controlling malarial parasite proliferation in the host, and the DNase that is released from the parasite may be a critical factor for NET destruction. In this study, we identify a DNase sequence in *Plasmodium falciparum* that contains a signal peptide and is homologous to bacterial TatD DNases (herein named TatD-like DNases) that are actively expressed in both *P. falciparum* and in rodent malarial parasites. The expression of this TatD-like DNase is associated with parasite virulence, and TatD-like DNase-specific antibodies are key elements in providing anti-plasmodium immunity.

## Results

### The plasmodial TatD possesses a conserved feature of DNases

**Figure 1:**
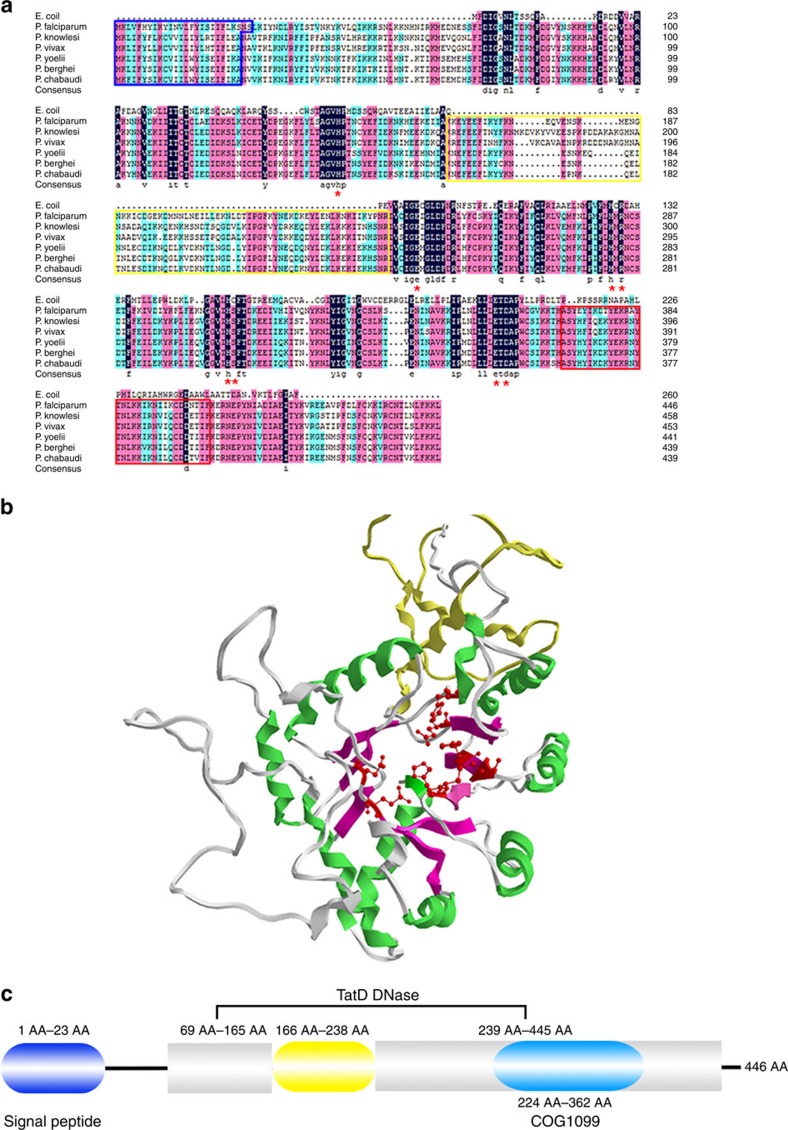
Sequence analysis and the predicted structure of PfTatD. (**a**) The TatD sequence alignment across various strains of *Plasmodium* and *E. coli*. The plasmodial TatD sequences contain a 24–26 AA signal peptide, whose cleavage site was between residues 26 and 27 in the *P. falciparum* or between 24 and 25 in other genera (blue box). A gap appeared in the predicted TatD DNase domain (yellow box) following a potent cell-binding residue (red box). The AAs that constitute the active site are indicated by red stars along the sequence. (**b**) The PfTatD 3D structure model was built using SWISS-MODEL. A template that contained 9 α-helices (green bands) and 8 β-pleated sheets (purple bands) in the secondary structure constituted a stable region. The yellow band indicates the gap in the TatD DNase domain. The red cylindrical bonds (red stars) indicate the conserved active site presented in **a**. (**c**) The schematic structural composition of the PfTatD domains. A 23-AA signal peptide site at the N terminus, followed by a 73-AA sequence gap (yellow rounded rectangle) that interrupted the TatD DNase domain, and AA residues from 224 to 362 indicated by the cerulean rounded rectangle.

Proteins of the TatD deoxyribonuclease family participate in many different biological processes, such as protein export and pathogenesis^18^,^19^. The sequence of *P. falciparum* (PfTatD) (PF3D7_0112000) is ∼30% similar to that of *Homo sapiens*, zebrafish, *Escherichia coli* and protozoa (Fig. 1a and Supplementary Fig. 1). The predicted PfTatD 3D model (without its signal peptide) contains a hydrolase active site surrounded by seven α-helices (Fig. 1b). The PfTatD domain contains a TIM barrel (COG 1099, multiple domain), which represents a nucleic acid binding region^20^ (Fig. 1c; cerulean, rounded rectangle).

Furthermore, compared to *E. coli* TatD, *Plasmodium* TatD contains an insertion in the middle of the sequence consisting of 73 amino acids (AAs) (Fig. 1a–c; yellow box, yellow tape and yellow rounded rectangle, respectively), which marks another feature of the *Plasmodium* TatDs that is distinct from other microbial homologues, apart from the signal peptide.

### The expression of TatD is associated with virulence

**Figure 2:**
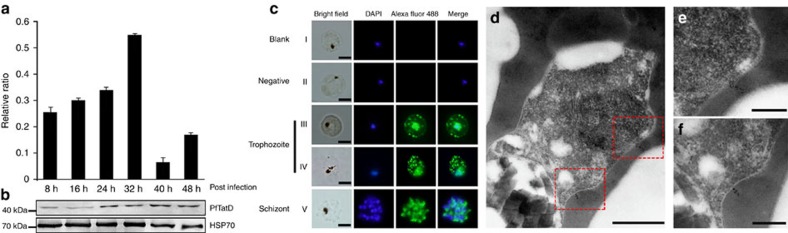
PfTatD is expressed at the mature stage of trophozoite-infected erythrocytes. (**a**) Transcriptional analysis of the PF3D7_0112000 gene was performed using quantitative PCR. Transcription was increased with parasite growth and reached its maximum at 32 h in the blood stage. The results are shown as the mean ±s.e.m. of 3 independent experiments. (**b**) An immunoblot was performed with rabbit anti-rPfTatD IgG (also see Supplementary Fig. 13). The top line indicates the expression of PfTatD every 8 h and the increased expression at 32 h in the blood stage. HSP70, presented in the bottom panel, was used as a control. (**c**) Indirect immunofluorescence of PfTatD was detected. The green fluorescence indicates that PfTatD was localized to the RBC membrane or the parasitophorous vacuole membrane of a mature trophozoite and was distinct from the nucleus in the schizont. The parasite nuclei were stained with DAPI. Scale bar, 5 μm. (**d**–**f**) Immune electron microscopy images of PfTatD. The gold particles were localized to the parasitophorous vacuole membrane, and there was a trend for localization to the periphery. Scale bar, (**d**) 500 nm and (**e**,**f**) 200 nm.

Transcription of PfTatD began early after parasite invasion into the erythrocytes, reaching the maximum level of transcription at 32 h and declining steeply at 40 h (Fig. 2a). The expression of the PfTatD protein began early after erythrocyte invasion and reached a maximal level 24 h post invasion, as shown by western blot analysis with a specific anti-rPfTatD antibody (Fig. 2b). Indirect immunofluorescence assays revealed that PfTatD was primarily localized to the periphery of infected erythrocytes at the mature trophozoite stage (Fig. 2c). Immunoelectron microscopy illustrated that PfTatD was primarily localized outside the parasitophorous membrane, with a trend towards localization at the red blood cell membrane (Fig. 2d–f). A similar pattern of expression was also observed in the infected red blood cells (iRBCs) of the *Plasmodium berghei* strain ANKA (Supplementary Fig. 2).

**Figure 3:**
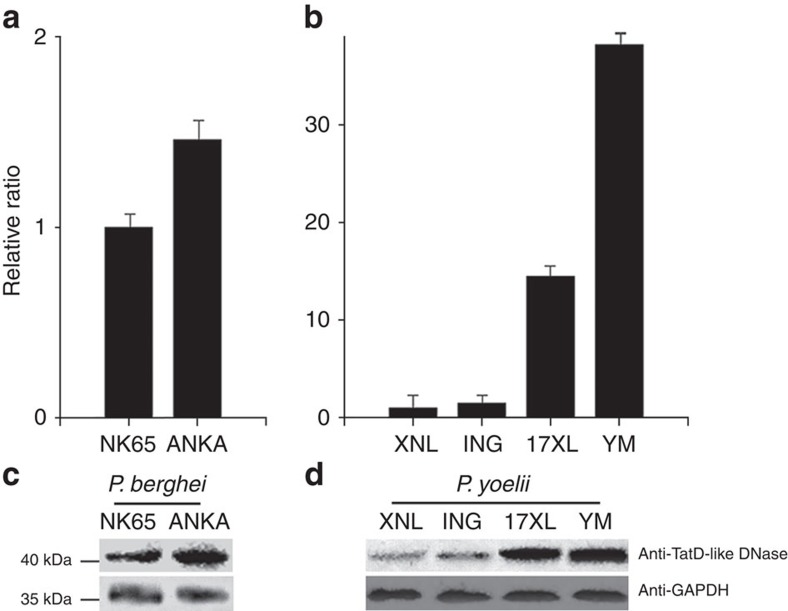
TatD-like DNase expression is associated with parasite virulence. Quantitative PCR and western blot assays were performed to measure mRNA and protein levels, respectively, of the TatD-like DNase in rodent malarial parasites. In the histograms presented in **a**,**b** parasite pathogenicity increased from the left to right, which positively correlated with transcription. Error bars represent the mean ±s.e.m. of three independent experiments. The expression patterns presented in **c**,**d** also positively correlated with the transcription results. The top panel indicates the TatD expression in different strains, which were collected at the same stage of the parasites. GAPDH is presented in the bottom panel and was used as a control. The full images of the western blot assays are shown in Supplementary Fig. 13.

To determine whether expression of the plasmodial TatD-like DNase is associated with pathogenicity or parasite proliferation within the host, we compared the expression of homologous proteins in several rodent parasite strains with lethality in mice. Transcription and expression of the *P. berghei* TatD-like DNase (PbTatD) were quantitatively higher in the virulent ANKA strain of *P. berghei* than the avirulent strain NK65 (Fig. 3a,c). This trend was also observed in strains of the *P. yoelii* parasite; specifically, the more virulent YM strain expressed more TatD than the avirulent XNL strain (Fig. 3b,d). Thus, the TatD-like protein is predominantly expressed in virulent parasite strains.

**Figure 4:**
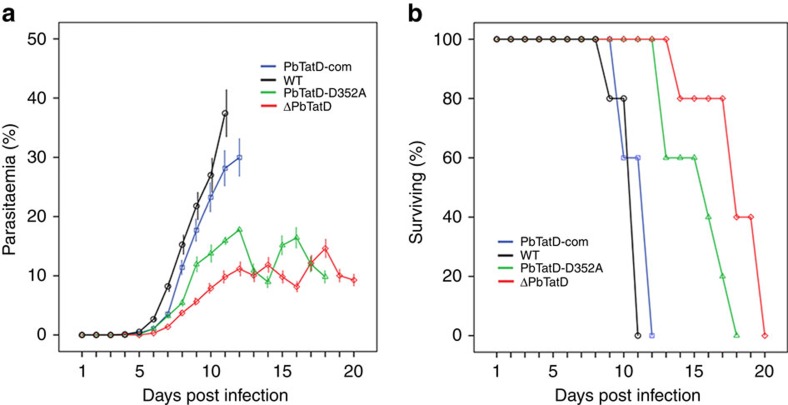
A comparison of the parasitaemia and survival of mice infected with WT and mutated strains. (**a**) A comparison of the mean parasitaemia with standard variations of the mice that were infected with WT *P. berghei*, ΔPbTatD, PbTatD-D352A and PbTatD-com parasites from day 1–20 after infection. The parasitaemia in mice that were infected with either ΔPbTatD (red line) or PbTatD-D352A (green line) was lower than in mice infected with WT (black line) or PbTatD-com parasites (blue line). The error bars indicate s.d. (**b**) The curves representing the survival rates of the mice that were infected with WT, ΔPbTatD, PbTatD-D352A and PbTatD-com strains. The mice that were infected with the WT exhibited 100% death 11 days after IP injection with 10^6^ infected RBCs. The PbTatD-com strain exhibited a similar result as WT, whereas the mice that were infected with the ΔPbTatD and PbTatD-D352A strains exhibited a twofold longer survival time.

In addition, gene knockout, complementation and mutation studies were performed to further confirm the hypothesis that TatD-like DNase is essential for parasite virulence. First, the TatD-like DNase gene of *P. berghei* ANKA was deleted using double-crossover method, a technique commonly applied in the functional analysis of malarial parasites^21^ (Supplementary Fig. 3a). The knockout strain (ΔPbTatD) was cloned and verified using both PCR with gene-specific primers and western blot assays (Supplementary Fig. 3b,c). The ability of the ΔPbTatD strain to proliferate *in vivo* was diminished compared with that of the wild-type (WT) strain (Fig. 4a,b). Second, the gene encoding PbTatD was re-introduced into the ΔPbTatD strain (named PbTatD-com), targeting the d-ssu-rrna gene with the selectable marker human dihydrofolate reductase (hDHFR) (Supplementary Fig. 4). Pathogenicity, in terms of proliferation rate and lethality, was fully restored in the knockout parasite after complementation with the PbTatD gene (Fig. 4a,b). Third, we introduced an aspartic acid-to-alanine mutation at the 352 position of PbTatD (named PbTatD-D352A) (Supplementary Fig. 5). The PbTatD-D352A mutation strain showed reduced proliferation and lethality in infected mice (Fig. 4a,b). Thus, the data collectively support our supposition that TatD-like DNase is a parasite virulence factor essential for the proliferation and pathogenicity of the parasite.

### Plasmodial TatD is a divalent metal-independent DNase

It was previously shown that the TatD of *E. coli* possesses DNA hydrolase activity^22^. We further characterized the DNA hydrolase activity of PfTatD by incubating the recombinant protein rPfTatD-GST (Supplementary Fig. 6) with human DNA in the presence of various divalent ions. First, rPfTatD-GST was able to hydrolyze double-stranded DNA in a concentration-dependent manner (Supplementary Fig. 7a), with maximal activity observed at 37 and 41 °C (Supplementary Fig. 8a). Such temperatures are not physiological but are commonly observed in infected patients. Because the activity of yeast and *E. coli* TatD is dependent on Mg^2+^ (refs 23, 24), we investigated the effect of divalent ions on the enzymatic activity of plasmodial TatD. Unexpectedly, the DNA hydrolase activity of rPfTatD was increased in the presence of higher concentrations of EDTA in the reaction buffer and, conversely, was inhibited by the addition of Mg^2+^, Ni^2+^ or other metals ions (Supplementary Figs. 7b,c and 8b). Furthermore, it has been reported that histidine and aspartic acid are critical for the structure and function of DNA hydrolases^25^. Four histidines and one aspartic acid residue were predicted in the sequences of plasmodial TatD hydrolases. To demonstrate that these two AAs are essential, we generated recombinant proteins containing mutations introduced at either the H303 or D352 position (Supplementary Fig. 5a) and tested their DNA hydrolytic activity. The results showed that none of these mutated proteins had DNase activity (Supplementary Fig. 5b,c). Thus, plasmodial TatD is a DNA deoxyribonuclease with a biochemical feature distinct from other DNA hydrolases.

### Plasmodial TatD hydrolyzes macrophage-derived ET

**Figure 5:**
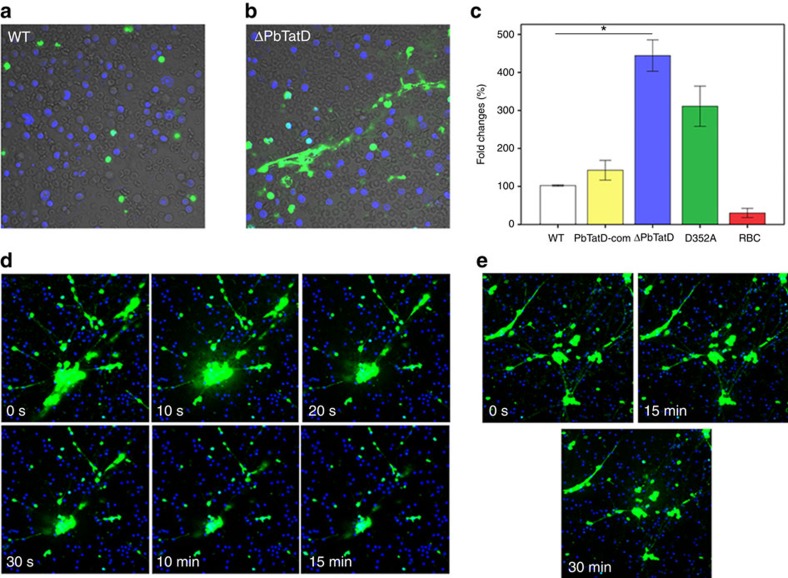
Formation of ETs after incubation of infected erythrocytes with macrophages. Representative images of the extracellular network structures after incubation of J774A.1 macrophages with WT *P. berghei* (**a**) and ΔPbTatD (**b**) infected erythrocytes. The ETs and dead cell nuclei were stained with Sytox green. Scale bar, 20 μm. (**c**) The relative quantification of ETs released by J774A.1 macrophages stimulated with the WT strain (white bar), ΔPbTatD strain (blue bar), PbTatD-com strain (yellow bar), PbTatD-D352A strain (green bar) or RBCs (red bar) are shown. The WT group was regarded as 100%. The results are the averages of three independent experiments (mean ±s.e.m., *P<0.05 in two-tailed Student's *t*-test). (**d**,**e**) Images of ETs after the addition of rPbTatD-GST or rPbTatD-D352A-GST in the cultivation at various time points. rPbTatD-GST showed DNase activity in a time-dependent manner (**d**), whereas rPbTatD-D352A-GST did not show any activity (**e**).

The involvement of parasite-infected erythrocytes in the induction of extracelluar trap (ET) structures *in vitro* was first tested by co-cultivation of J774A.1 macrophages with RBCs that were infected with either WT *P. berghei*, the ΔPbTatD strain, the PbTatD-D352A mutant strain or the PbTatD-com strain. Both the ΔPbTatD and PbTatD-D352A mutant strains induced ET network formation *in vitro*, as is commonly observed in bacterial pathogens after incubation with macrophages, but there were significantly fewer ET nets induced by RBCs infected with WT *P. berghei* and the PbTatD-com strain (Fig. 5a–c, *P*<0.05 in two-tailed Student's *t*-test). Similarly, mouse neutrophils could generate ET when co-cultivated with the ΔPbTatD strain, but they could not produce ET in the presence of WT parasites (Supplementary Fig. 9). Thus, WT *P. berghei* ANKA inhibits ET formation in a PbTatD-dependent manner, which supports our hypothesis that TatD-like DNase is released from parasite-infected RBCs and mediates the interaction of the parasite with the host innate immune system^10^,^26^.

To further confirm the role of the TatD enzyme in the interaction of plasmodial parasites with host immune cells, recombinant proteins of either rPbTatD-GST or rPbTatD-D352A-GST were added into ΔPbTatD strain/macrophage cultures. Although rPbTatD-GST could hydrolyze ET structures in a time-dependent manner, the mutated protein showed no function in DNA hydrolysis at any time point in the assays (Fig. 5d,e). Thus, the data collectively support the hypothesis that plasmodial TatDs are parasite virulence factors that may be released from infected erythrocytes to hydrolyze the DNA component in ET structures formed by immune cells.

### Immunization of recombinant TatD protects against infection

**Figure 6:**
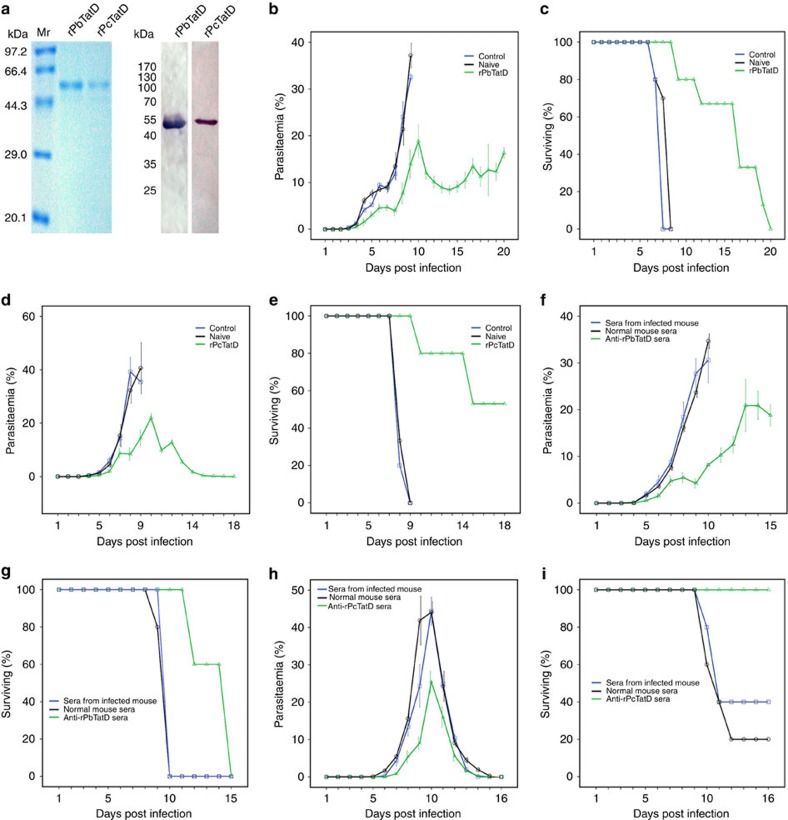
Immunization with the TatD-like DNase generates protective immunity against rodent malaria. (**a**) rPbTatD-HIS and rPcTatD-HIS were purified by affinity chromatography using His GraviTrap. The SDS–PAGE and western blotting assays revealed that the recombinant proteins were >90% pure. (**b**) BALB/c mice immunized with Freund's adjuvant alone or without any immunization (Naive) exhibited 3.12-fold higher parasitaemia than the rPbTatD-immunized group on day 11 post infection; the error bars indicate the s.d. (**c**) Mice in the rPbTatD-immunized group survived 10 days longer than the two control groups. (**d**,**e**) BALB/c mice immunized with Freund's adjuvant alone and naive mice exhibited 4.66-fold higher parasitaemia than the rPcTatD-immunized group on day 8 post challenge; the error bars indicate the s.d. All of the mice that received Freund's adjuvant alone and the naive group died on day 9 post infection; however, eight of the mice in the rPbTatD-immunized group were completely protected. (**f**,**g**) The group that received serum from a previously infected mouse exhibited 3.97-fold higher parasitaemia than the group that received anti-rPbTatD serum on day 10 post infection. Compared with the other groups, the group that received anti-rPbTatD serum survived for 5 days longer; the error bars indicate the s.d. (**h**,**i**) The group that received serum from a previously infected mouse exhibited 1.79-fold higher parasitaemia than the group that received rPcTatD-specific serum on day 10 post infection. The mice that received rPcTatD-specific serum showed a 100% survival rate. The error bars indicate the s.d.

We further investigated the significance of TatD-specific antibodies in host protection against parasite infection *in vivo*. BALB/c mice (*N*=15 in each group) were immunized four times with the His-tagged recombinant *P. berghei* TatD-like DNase protein (rPbTatD-HIS) (Fig. 6a) and Freund's adjuvant. As expected, a reduction in parasitaemia and delayed death was observed in the immunized group after challenge by intraperitoneal (IP) injection with 10^6^ iRBCs (Fig. 6b,c). Furthermore, there was a reduction in parasitaemia and protection in infected mice passively treated with intravenous (IV) injection of anti-rPbTatD sera (Fig. 6f,g). Moreover, immunization with the His-tagged recombinant TatD-like DNase of *P. chabaudi* (rPcTatD-HIS) produced similar results (Fig. 6d,e,h,i). The experiment was repeated with C57BL/6 mice, and similar results were obtained (Supplementary Fig. 10). Cross species protection was tested. Mice were immunized with rPbTatD-HIS and challenged with either the *P. chabaudi* AS or *P. yoelii* 17XL strain. A reduction in parasitaemia and delayed death was observed in the immunized group (Supplementary Fig. 11). Moreover, plasmodial TatD-specific antibodies showed very minimal cross-reaction with host proteins of various organs (Supplementary Fig. 12). These data collectively indicate that TatD-specific antibodies provide protection against parasite infection.

## Discussion

There are diverse mechanisms by which malaria parasites escape host innate immunity^27^, but the proteins involved in these processes are poorly understood. These molecules are likely to be important virulence factors that can be explored as potential candidates for vaccine development or novel therapies.

In this study, a gene encoding the *E. coli* TatD-like homologous protein was identified in every genome of plasmodial parasites. Bioinformatics analysis showed that the TatD sequences of *Plasmodium*, including *P. falciparum*, *P. vivax*, *P. knowlesi*, *P. chabaudi*, *P. yoelii* and *P. berghei*, shared more than 80% identity, which indicates a conserved function for this protein in the *Plasmodium* genus (Fig. 1). All plasmodial TatD sequences contained a conserved motif with the 21 AA sequence characteristic of the TatD deoxyribonuclease family^22^. In addition to the conserved deoxyribonuclease motif, a signal peptide was exclusively found in the N termini of all of *Plasmodium* TatD proteins and was absent in other apicomplexan parasites and metazoans (Fig. 1). The indirect immunofluorescence and electron microscopy results demonstrated that TatD-like DNase is expressed in the blood stage of plasmodial parasites and is likely secreted during parasite maturation. The electron microscopy results showed that the protein is transported towards the erythrocyte membrane, which is consistent with the bioinformatics analysis. PfTatD does not contain the Pexel motif^28^ frequently identified in the membrane protein, which indicated that this class of proteins follows the classical pathway of transportation.

The possible association of TatD expression with parasite virulence was investigated by various approaches, including real-time PCR (rtPCR), western blotting, gene knockout and complementation. The expression of TatD was increased in the virulent *P. bergeri* ANKA strain compared with the avirulent strain (*P. bergeri* NK65). Similar data were obtained when TatD expression was assessed in the *P. yoelii* strains (Fig. 3). Using recombinant proteins, we confirmed that plasmodial TatD is critical for DNA hydrolysis. Unlike other DNases, such as DNase II, the DNA hydrolytic activity of plasmodial TatD is not divalent metal-dependent (Supplementary Fig. 7).

It has been well established that that host macrophages and neutrophils release ETs to confine invading pathogens on activation. Some of the pathogens release DNase to counteract the trap. The trap structure has been observed in skin blood vessels of children with uncomplicated malaria^14^. Furthermore, *Toxoplasma gondii*, another Apicomplexan, can induce neutrophils to release NETs directly^29^. In the current experiment, only the TatD knockout and TatD mutated parasites, and not the WT strain, were able to induce ET. In addition, ETs were immediately destroyed on addition of recombinant TatD to the culture. Thus, the data support the conclusion that plasmodial parasites use TatD to degrade ET structures to escape host immune clearance.

The sequence conservation and significant DNA hydrolytic activity of plasmodial TatD led us to further explore the possibility of using this molecule as an immunogen for vaccination. Surprisingly, we observed an increase in protection in two mouse models (both C57BL/6 and BALB/c) (Fig. 6; Supplementary Figs. 10 and 11). These data indicate that the plasmodial TatD protein should be further explored as a potential candidate in malaria vaccine development.

In conclusion, we identified a TatD-like DNase that is present in every *Plasmodium* species and has biochemical features distinct from those of the bacterial TatD family. The enzyme was predominantly expressed in mature asexual-stage parasites but may also be expressed at other stages^30^. The expression of this enzyme was associated with parasite virulence and was necessary for successful proliferation in the host. Moreover, antibodies against plasmodial TatD provided protection against parasite infection, which supports the use of *P. falciparum* TatD-like DNase as a potential candidate for malarial vaccine development.

## Methods

### Parasites

The *P. falciparum* 3D7 strain was cultured and synchronized *in vitro* as previously described^31^. Briefly, parasites were continuously cultured in MCM culture medium in candle gar and incubated at 37 °C. The synchronization of parasites was performed by incubating the cultures in 5% (w/v) D-sorbitol (Sigma, USA), after which the cells were washed twice with MCM before transferring to new flasks.

*P. berghei* (strains ANKA and NK65), *P. chabaudi* (strain AS) and *P. yoelii* (strains YM, XNL, ING and 17XL) were maintained through infection of C57BL/6 mice by IP injection of 1 × 10^6^ parasitized erythrocytes as previously described^32^. Parasitaemia was monitored by examining Giemsa-stained thin blood smears under a light microscope.

Six- to eight-week-old BALB/c and C57BL/6 female mice were purchased from Jilin University Experimental Animal Center (Changchun, China). All mice were maintained in an enriched environment, and all experiments were carried out in accordance with the guidelines established by Jilin University and were approved by the Animal Welfare and Research Ethics Committee of Jilin University. The ethical clearance number for this study is KZ2010-01.

### *Plasmodium* TatD-like DNase sequence analyses

The putative TatD nuclease sequences of *P. falciparum*, *P. berghei*, *P. knowlesi*, *P. vivax*, *P. chabaudi*, *P. yoelii*, *Babesia equi*, *Cryptosporidium muris*, *Trypanosoma brucei*, *Theileria parva*, *Neospora caninum*, *Leishmania*, *Homo sapiens*, *E. coli* and *Schistosoma mansoni* were compared by a multiple alignment constructed with DNAMAN 7 (Lynnon Corporation, USA). To assess the extent of the diversity and evolutionary relationships among the TatD nucleases (Gene IDs for phylogenies and comparisons are shown in Supplementary Table 1), the MEGA 5.2 program^33^ was used to construct a phylogenetic tree on the basis of the evolutionary distances calculated by the Kimura two-parameter model^34^.

The putative function of PfTatD was identified by GO analysis (http://geneontology.org/). The signal peptide was predicted using the SignalP 4.1 server^35^. The PfTatD 3D model was built using SWISS-MODEL^36^,^37^,^38^.

### Total RNA extraction and quantitative rtPCR

Total RNA was extracted from synchronized parasites every 8 h throughout the entire life cycle as previously described^39^. The rodent parasites were collected at a mixed stage when parasitaemia reached ∼5%. rtPCR was performed in triplicate using the FastStart Universal SYBR Green Master (Roche) with specific primers (Supplementary Table 2) and an Applied Biosystems 7500 rtPCR system. The data were analysed using the comparative critical threshold (ΔΔCt) method, in which the amount of target RNA was compared to an internal control, seryl-tRNA synthetase (PF3D7_1205100)^40^. Rodent malarial TatD transcription was evaluated using the same method.

### Expression of recombinant proteins and antibody generation

Genes encoding the TatD-like DNase of *P. falciparum*, *P. berghei* and *P. chabaudi* were cloned from the corresponding parasites using the rtPCR method. The sequences coding for the H303 and D352 mutations in PbTatD were chemically synthesized, and all genes were cloned into the pET-28a and pGEX-4T-1 vectors, respectively (Invitrogen). HIS-tagged and GST-tagged recombinant proteins were expressed in BL21(DE3) cells using the pET-28a and pGEX-4T-1 vectors, respectively, and the soluble proteins were purified using His GraviTrap and Glutathione Sepharose (GE Healthcare), respectively, according to the manufacturer's instructions. Anti-rPfTatD/rPbTatD/rPcTatD sera were raised in New Zealand white rabbits by four immunizations with 300 μg of recombinant protein and Freund's adjuvant. The sera were collected following titre evaluation. Specific IgGs were purified from the sera using Protein A (GE Healthcare) according to the manufacturer's instructions.

### Characterization of DNase activity in recombinant PfTatD

Human whole-blood cells were obtained from the blood bank of Changchun City, and the experiments with human genetic samples were approved by the Ethical Committee of Jilin University (the ethical clearance number for this study is KZ2010-01). Genomic DNA was extracted from whole-blood cells using the QIAamp DNA Mini Kit (Qiagen) according to the manufacturer's protocol. The DNA was incubated with 30, 15, 7.5, 3 or 1.5 μM recombinant PfTatD in Tris–HCl buffer (pH 7.5) in a final volume of 40 μl at 37 °C. To search for the optimal enzymatic temperature, digestion reactions were performed at 16, 25, 35, 37 and 41 °C. To determine the effect of metallic ions on nuclease activity, different concentrations of EDTA, Mg^2+^, Ni^2+^ and other bivalent metal ions were added to the reaction. The results were visualized on 1.2% agarose gels under UV illumination following ethidium bromide staining.

### Parasite protein preparation and western blotting analysis

The proteins from synchronized *P. falciparum*-iRBCs were run on an SDS–PAGE gel and analysed via western blotting. Briefly, the proteins were electrophoresed on 12% SDS–PAGE gels and then transferred to 0.2-μm nitrocellulose membranes using the Semi-Dry blotting system (Bio-Rad, CA, USA). A rabbit polyclonal anti-rPfTatD (1:1,000) IgG was used as the primary antibody, and either an AP-labelled goat anti-rabbit IgG (1:20,000) (Abcam, Shanghai, China) or an HRP-labelled goat anti-rabbit IgG (1:6,000) (Abcam, Shanghai, China) was used as the secondary antibody. Rodent parasite-infected erythrocytes were collected at 5% parasitaemia for every strain. The samples were analysed as described above with the corresponding specific primary antibody. The host proteins used in the cross-reactivity assay were prepared by grinding them with liquid nitrogen, and the primary antibody used was a mouse anti-rPbTatD IgG. A GAPDH monoclonal antibody (Abcam) and rabbit anti-HSP70 polyclonal antibodies (Antibodies-online, Germany) were used in a corresponding experiment, and either the AP-labelled goat anti-mouse IgG (1:20,000) or the HRP-labelled goat anti-rabbit IgG (1:5,000) was used as the secondary antibody. Full versions of all blots are provided in Supplementary Fig. 13. 

### Immunofluorescence assay

The air-dried thin smears of blood cells infected with parasites at different stages were fixed with 4% paraformaldehyde and 0.0025% glutaraldehyde for 30 min at room temperature and were then blocked with 5% non-fat milk. The samples were incubated with a rabbit anti-TatD-specific IgG (1:100) for 60 min at 37 °C, followed by staining with a goat anti-rabbit IgG (1:600) (Alexa Fluor 488) for 60 min at 37 °C. The nuclei were stained with 4,6-diamidino-2-phenylindole (DAPI). The images were captured using a fluorescence microscope (Olympus, BX 53).

### Immunoelectron microscopy assay

For immunoelectron microscopy assay, mature trophozoite-infected erythrocytes (*P. falciparum* 3D7 and *P. berghei* ANKA) were fixed with 0.2% v/v glutaraldehyde and 1% w/v paraformaldehyde in PBS for 30 min at 4 °C. The samples were embedded in LR white resin (Sigma) at 50 °C for 24 h after dehydration. Thin sections were mounted on nickel grids. For immunostaining, samples were incubated with a TatD-like DNase-specific rabbit polyclonal IgG (1:100) at 4 °C overnight after blocking with 3% non-fat milk, followed by incubation with a goat anti-rabbit IgG conjugated to 5 nm gold (1:40) (Sigma G7277) at 37 °C for 60 min. The IgG from pre-immune sera was used as the primary antibody control. The samples were examined with a transmission electron microscope (Hitachi H-7650, Japan).

### DNA constructs and parasite transfection

We chose the double-crossover method to generate a TatD knockout as previously described^41^. Briefly, as presented in Supplementary Fig. 3a, the plasmid was built with pl0001 (MR4, ATCC, USA). Two homology sequences from the PbTatD 5′ and 3′ Untranslated regions (UTRs) were PCR-amplified and inserted upstream and downstream of the DHFR gene, respectively. A total of 20 μg of linear plasmid (digested with ApaI and NotI) was mixed with 10^7^ schizonts purified with 55% Nycodenz (AXIS-SHIELD). The mixture was pulsed at 0.8 kV and at a capacitance of 25 μF using a Gene Pulser II (Bio-Rad) and then IV injected into one C57BL/6 mouse. The parasites were selected *in vivo* by the addition of a pyrimethamine solution to the drinking water 24 h after injection. A PCR method with primers specific for the insert was used to verify the transfection and the gene knockout. The PbTatD knockout strain parasites (ΔPbTatD) were cloned using a limiting-dilution method.

To generate a parasite strain with a reconstructed TatD gene in the ΔPbTatD strain, the plasmid pL0017 (MR4, ATCC, USA) was used as a backbone for the generation of the PbTatD complementation construct and to target the c- or d-ssu-rrna sequence for integration by single crossover recombination. Briefly, the hDHFR selection marker was amplified from a PYC plasmid (a gift from Professor Jing Yuan, Ximen University) and replaced the GFPM3 (BamHI/XbaI) in pL0017 to build pL0017-hDHFR. Then, the PbTatD-containing 3′ UTR was amplified from the genome and exchanged the GFPM3 (BamHI/XbaI) in a new pL0017 backbone to build pL0017-TatD. Pbeef1aa+PbTatD+3′ UTR was amplified from pL0017-TatD and replaced the DHFR-TS cassette (HindIII/EcoRV) in pL0017-hDHFR to obtain the PbTatD complementation plasmid (PbTatD-com). The PbTatD-com plasmid was linearized with SacII and transfected into the ΔPbTatD parasites as previously described. The transfected parasites were selected after a 4-day-IV treatment with the drug WR99210 (Sigma). The correct integration of the complementation construct into the d-ssu-rrna on chromosome 5 was verified by PCR using specific primers (Supplementary Table 2). Expression of the PbTatD gene in PbTatD-com parasites was analysed by western blot analysis using an anti-rPbTatD IgG as the primary antibody and glyceraldehyde-3-phosphate dehydrogenase (GAPDH) as the control.

To generate a parasite strain with a mutation at the 352 position, we used the pl0001 plasmid to generate the allelic replacement construct of PbTatD-D352A. The fragment (–1,500 to –1,000 bp) was amplified and inserted at the 5′ end of the DHFR-TS cassette. The fragment (–1,000 to +1,389 bp with mutation) was chemically synthesized by Sangon Biotech (Shanghai Co., Ltd) and was inserted at the 3′ end of the DHFR-TS cassette, as illustrated in Supplementary Fig. 5. The sequences of the primers used for this study are listed in Supplementary Table 2.

### ET formation assay *in vitro*

J774A.1 macrophages were purchased from the Cell Resource Center, IBMS, China and were cultivated in DMEM on poly-L-lysine-coated glass-bottom dishes with a confluence of 1 × 10^5^ cells/well. A total of 10^6^ mouse red blood cells (5% parasitaemia) infected with either the WT *P. berghei* ANKA or ΔPbTatD strain were added to the dishes and cultivated for 6 h. A total of 10^6^ normal mouse red blood cells were used as the control. After co-cultivation, the medium was removed, and the cells were stained with Sytox green (a cell-impermeable dye) and DAPI (a cell-permeable dye) for 5 min without fixation and then imaged immediately for the presence of NETs via confocal laser microscopy. In some of the experiments, rPbTatD-D352A-GST and rPbTatD-GST were added after staining. ETs were counted in 50 fields when the length of extracellular DNA was over 50 μm. Three independent experiments were performed.

Mouse neutrophils were purified using Percoll as previously described^29^. Briefly, mouse blood was collected, washed and resuspended in RPMI. The cells were layered onto a three-layer Percoll gradient of 55, 65 and 80% Percoll and centrifuged at 500*g* for 30 min at 4 °C. The neutrophils (65/80% interface) were collected, washed twice with PBS, resuspended in RPMI and counted. The neutrophils were incubated with different *P. berghei* ANKA strains for 2 h.

### Immunization with rPbTatD and rPcTatD proteins

For the first immunization, 50 μg/mouse (BALB/c or C57BL/6 strain, female, six- to eight-week-old) rPbTatD-HIS or rPcTatD-HIS emulsified with complete Freund's adjuvant was intramuscular injected, followed by three immunizations with 50 μg recombinant protein emulsified with incomplete Freund's adjuvant with 2-week inter-injection intervals. The mice in the control group were injected with Freund's adjuvant only. The antibody titres were evaluated by ELISA. When the titre exceeded 1:32,000, ∼10^6^ iRBCs per mouse were IP injected in all groups. The parasitaemia was measured daily by counting 3,000 cells per Giemsa-stained blood smear.

### Passive immunization assays with anti-rPbTatD/rPcTatD sera

In the passive immunization assays, each mouse was IV injected with 500 μl of sera from either an rPbTatD/rPcTatD-immunized mouse or a mouse previously infected with *P. berghei* ANKA or *P. chabaudi* AS 24 h earlier. After challenging with 10^5^ iRBCs, the parasitaemia was measured and counted as described above.

## Additional information

**How to cite this article:** Chang, Z. *et al*. The TatD-like DNase of *Plasmodium* is a virulence factor and a potential malaria vaccine candidate. *Nat. Commun.* 7:11537 doi: 10.1038/ncomms11537 (2016).

## Supplementary Material

Supplementary Information Supplementary figures 1-13, Supplementary tables 1-2
