# Supplementary material for: The TatD-like DNase of Plasmodium is a virulence factor and a potential malaria vaccine candidate
Source: Nat Commun. 2016 May 6;7:11537. doi: 10.1038/ncomms11537 (PMC4859065; doi:10.1038/ncomms11537)
Supplement: Supplementary Information — Supplementary figures 1-13, Supplementary tables 1-2 [file ncomms11537-s1.pdf]

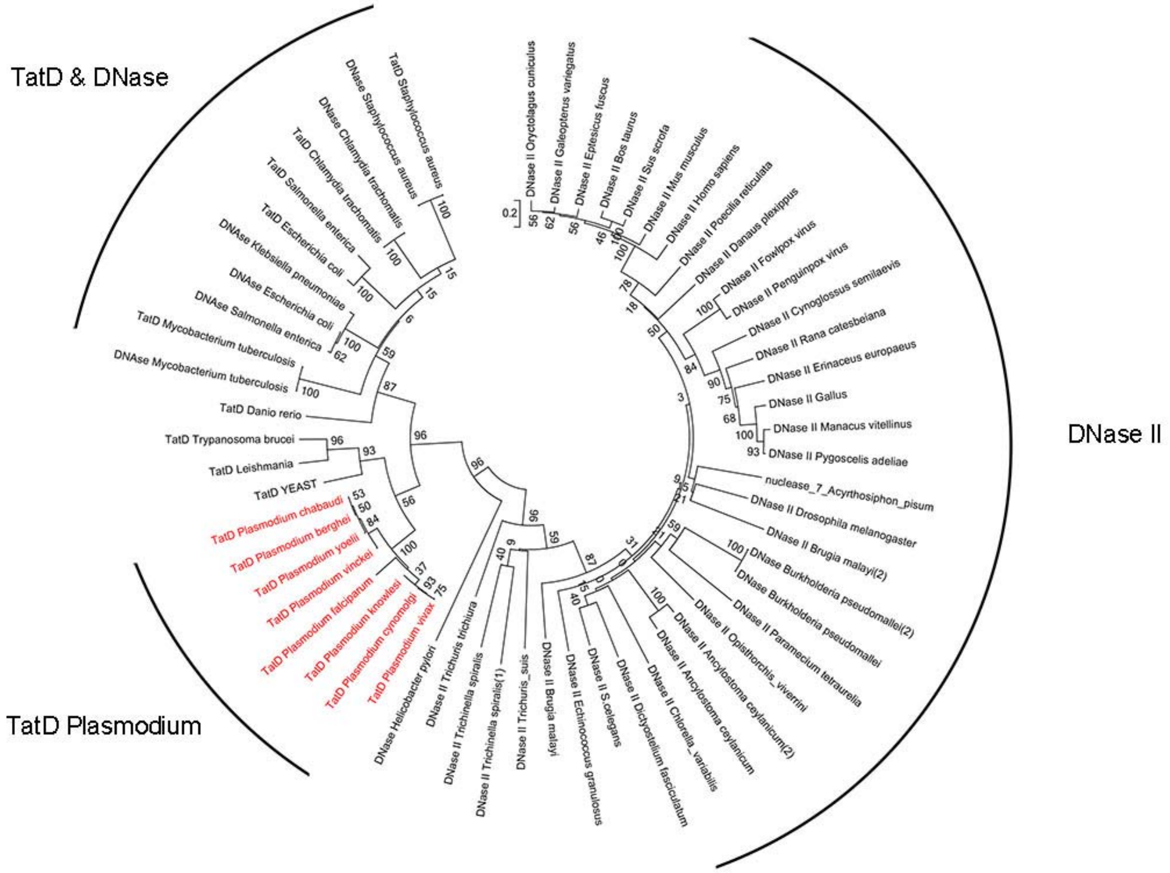

Supplementary Figure 1| Phylogenetic relationships of the TatD and DNase II sequences. A phylogenetic tree of the putative *Plasmodium* and selected apicomplexan TatD nucleases and DNase II nucleases from other species, as inferred using a minimum-evolution tree, was built based on the evolutionary distances calculated using the Kimura two-parameter method.

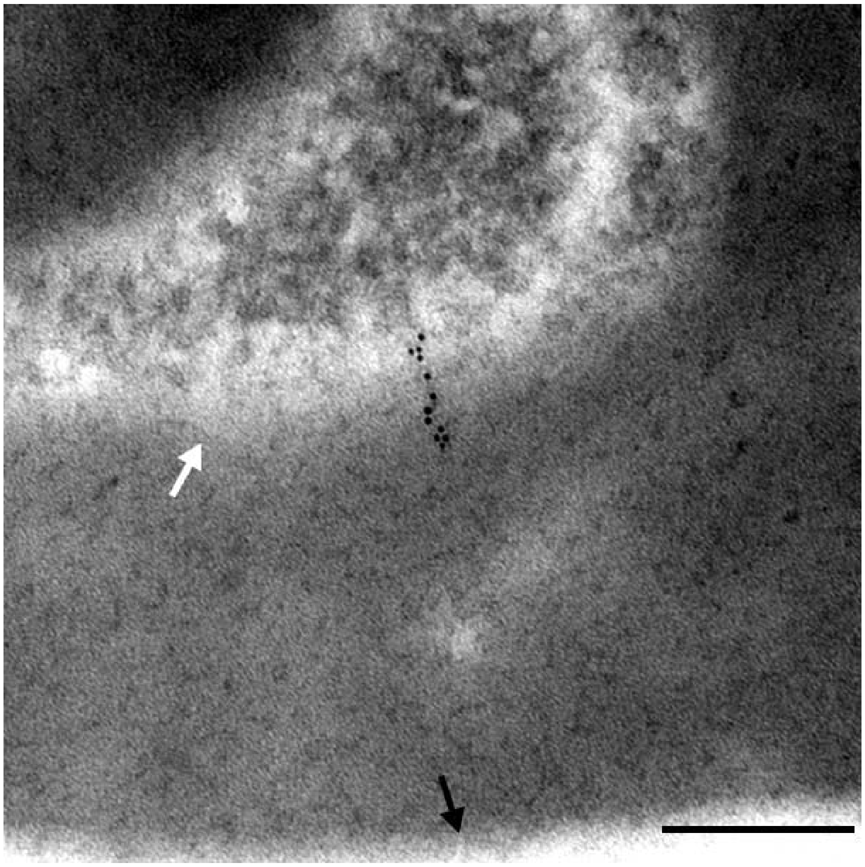

Supplementary Figure 2 | Detection of *P. berghei* ANKA TatD by immunoelectron microscopy. The white arrow indicates the parasitophorous vacuole membrane, and the black arrow indicates the blood cell membrane. The gold particles were localized to the parasitophorous vacuole membrane, and they localized towards the periphery. Scale bar, 200 nm.

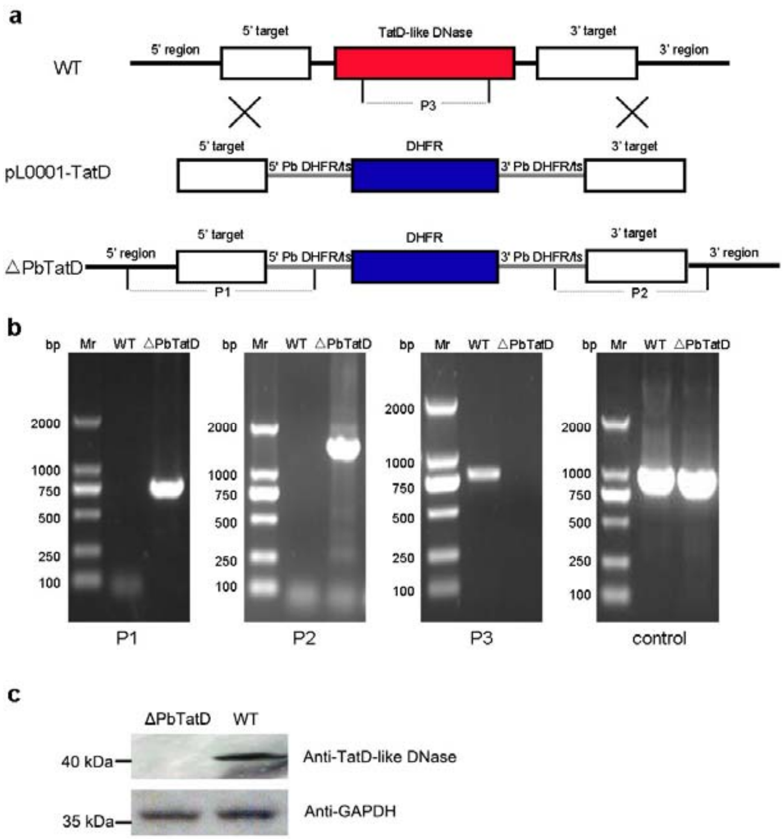

Supplementary Figure 3 | Gene disruption of the TatD homologous gene in *P. bergh ei*. (a) Schematic representation of the approach used for gene deletion by double-cross o f the gene encoding PbTatD. The 5' and 3' UTR regions were selected as homology seq uences for genetic crossing. P1, P2 and P3 are the primer regions used to identify the in tegration. (b) Verification of the correct integration of the vector in the *P. berghei* geno me was achieved by PCR. The location of the primers and the product sizes are indicate d in (a). P1: verification of 5' integration; P2: verification of 3' integration; P3: verificat ion of PbTatD replacement; Control: verification of DNA quantity. (c) The expression of PbTatD after gene deletion is shown by immunoblot. The top panel indicates the expres sion of PfTatD in the WT strain but not in the ΔPbTatD strain; GAPDH, as shown in t

he bottom panel, was used as the control. Full images for panel (c) are shown in Supple mentary Fig. 13.

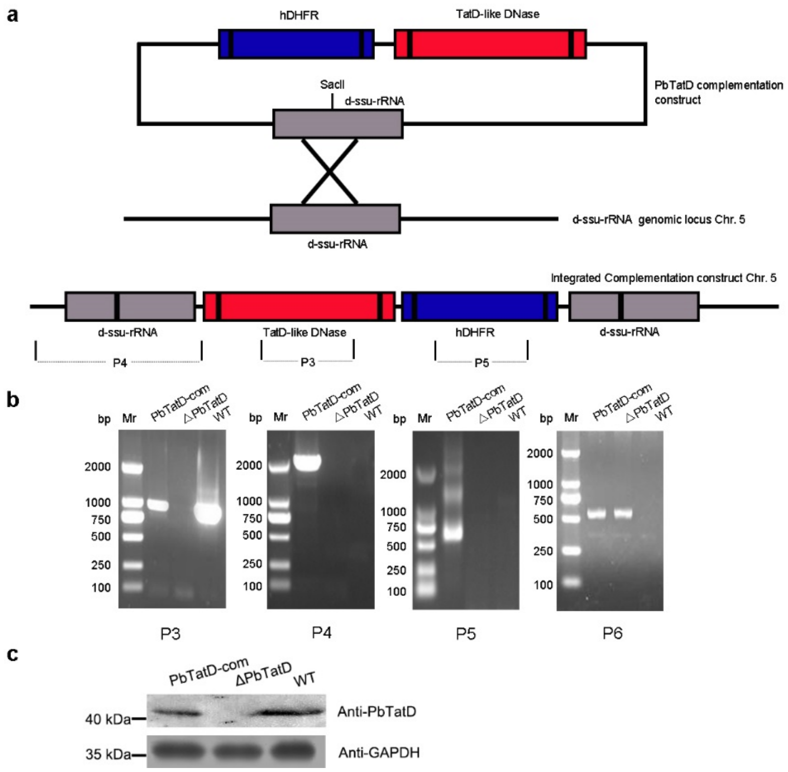

Supplementary Figure 4 | Genetic complementation of ΔPbTatD parasites with wild type PbTatD.

(a) Diagrammatic representation of the construct (PbTatD-com) used for gene complement ation. The d-ssu-rrna genomic locus in Chr.5 was used for integration. P3, P4, and P5 ar e the primers used to identify the integration. (b) The integration verification of PbTatD-com was achieved by PCR. The location of the primers are indicated in (a). P3: verifica tion of PbTatD; P4: verification of the d-ssu-rrna integration; P5: verification of hDHFR; P6: verification of DHFR-TS. (c) Western blotting analysis shows the expression of PbT atD in the wild type and PbTatD-com strains and the absence of PbTatD in the ΔPbTat D strain. The full images for panel (c) are shown in Supplementary Fig. 13.

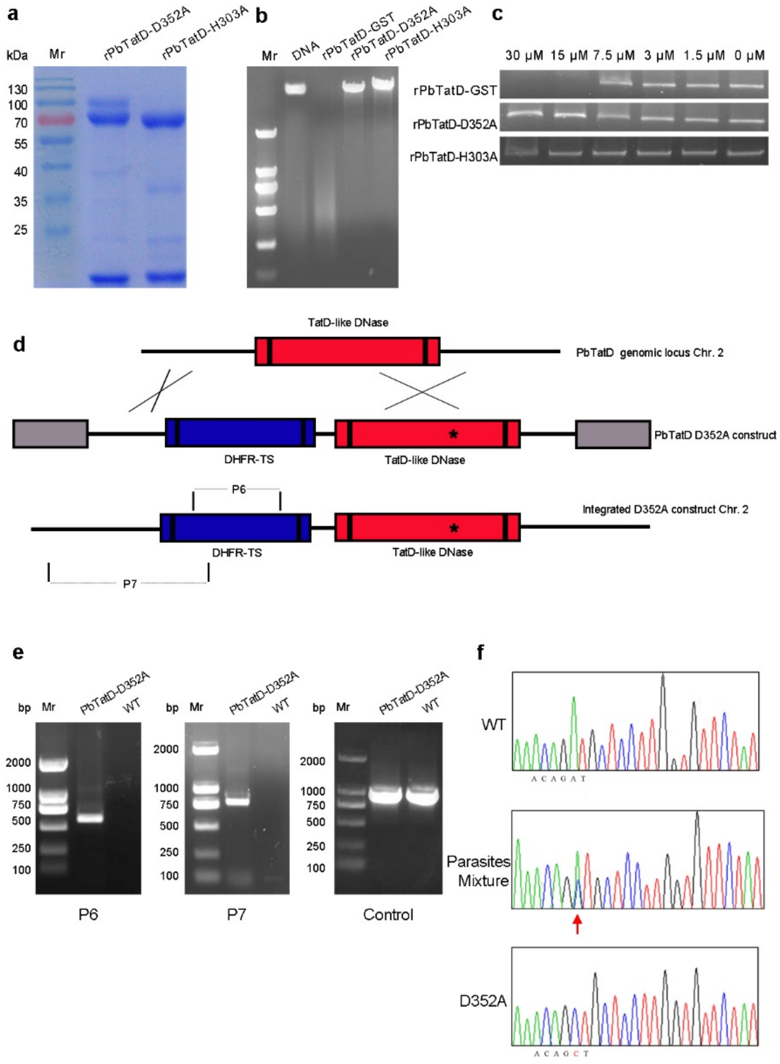

Supplementary Figure 5 | Generation of parasites with PbTatD-D352A substitutions.

(a) The purified recombinant proteins rPbTatD-D352A and rPbTatD-H303A are represented. In (b) and (c), the DNA hydrolytic activity of the WT rPbTatD-GST protein, rPbTatD-D352A and rPbTatD-H303A are illustrated. The WT rPbTatD-GST was the only one with DNA hydrolytic activity. (d) Schematic construct for the PbTatD nucleotide replacement is shown. A diagram depicting the PbTatD genomic locus (Top), the PbTatD-D352A replacement vector (centre), and the PbTatD-D352A mutant parasite locus (bottom) are shown. (e) Verification of the correct replacement was achieved by PCR analysis. P3: verification of the PbTatD; P4: verification of the

d-ssu-rrna integration; P5: verification of the hDHFR; P6: verification of the DHFR-TS. P6: verification of the DHFR-TS; P7: verification of 5' integration; Control: verification of DNA quantity. (f) DNA sequencing confirmed the allelic replacement. The top panel shows the nucleotide sequence from the WT strain ANKA, the middle panel shows the double peak (red arrowed) of the targeted nucleotide from transfected parasite mixtures, and the bottom panel shows the targeted mutations of the clone PbTatD-D352A.

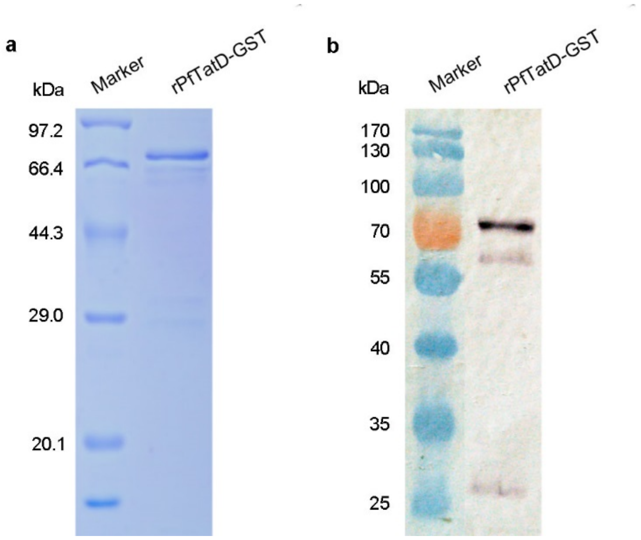

Supplementary Figure 6 | Purification and confirmation of recombinant rPfTatD-GST. rPfTatD-GST has a molecular weight of 71 kDa and was expressed in *E. coli,* purified and analysed by SDS-PAGE (a). The protein was further confirmed by Western blotting using an anti-GST-tag mAb (b).

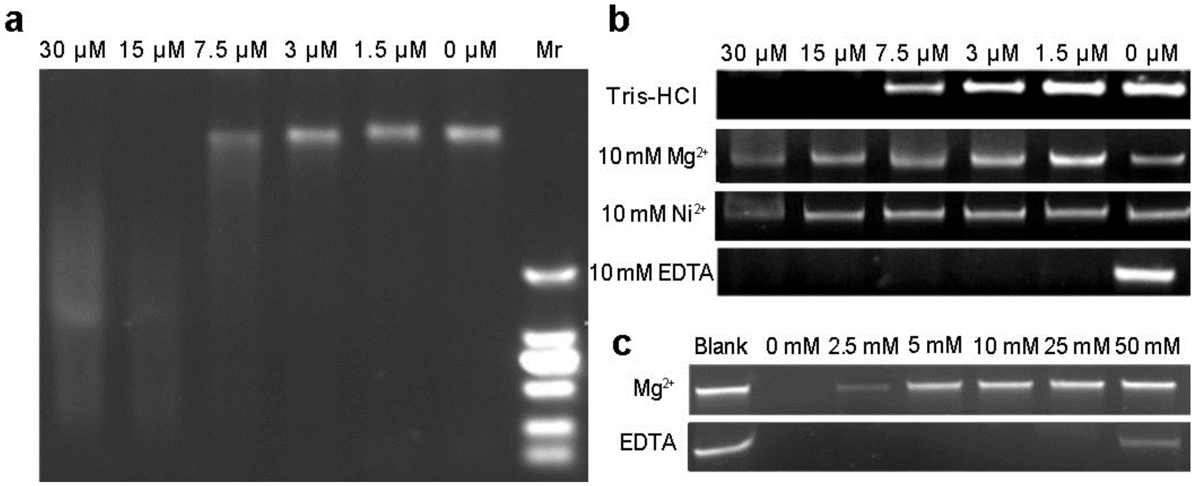

Supplementary Figure 7 | The DNA catalytic activity of PfTatD is ion-independent. (a) rPfTatD-GST at a concentration of 0 to 30 μM was incubated with 20 ng of human DNA in 50 mM Tris-HCl (pH 7.5) at 37°C for 30 min. The human DNA was completely hydrolysed at 15 μM. (b) 10 mM Mg^{2+} and Ni^{2+} were added to the reaction, and the hydrolysis was greatly inhibited compared to the blank control. By contrast, the addition of 10 mM EDTA enhanced the activity, as shown in the fourth line. (c) The hydrolysis was significantly reduced with increasing Mg^{2+} concentrations added to the reaction buffer, whereas the EDTA exerted no influence on hydrolysis until its concentration reached 50 mM.

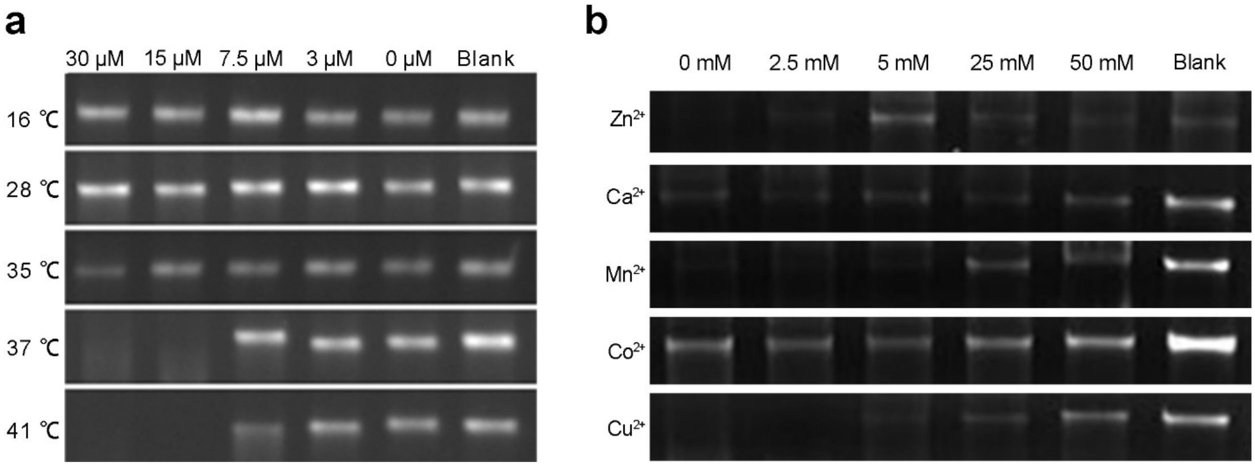

Supplementary Figure 8 | Analyses for the optimal reaction temperature and metal ion-dependency of PfTatD. (a) The human DNA was digested with PfTatD at concentrations of 0 to 30 μM and at temperatures of 16 to 41°C. At 37°C, the protein began to exhibit nuclease activity. (b) Zn^{2+}, Ca^{2+}, Mn^{2+}, Co^{2+} or Cu^{2+} was added to the reaction with 10 μM rPfTatD, which is unable to fully digest DNA at 37°C for 30 min. As the ion concentration increased, the activity declined.

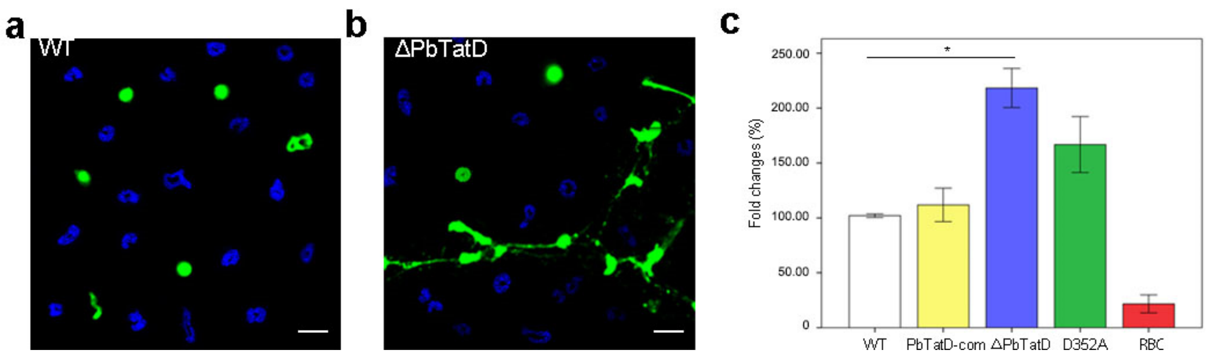

Supplementary Figure 9 |ΔPbTatD stimulates NET formation *in vitro*.

(a) Representative images of WT and (b) ΔPbTatD strains co-cultivated with neutrophils *in vitro*. The NETs and dead cell nuclei were stained with Sytox green, and all of the nuclei in the field were stained with DAPI (in blue). The scale bar is 10 μm. (c) The relative quantification of NETs that were released by neutrophils stimulated with the WT strain (white bar), the PbTatD-com strain (yellow bar), the ΔPbTatD strain (blue bar), the PbTatD-D352A strain (green bar) or the RBCs (red bar). The WT strain group was regarded as 100%. The results are the averages of 4 independent experiments (mean±SEM, *p<0.05 by two-tailed Student's t-test).

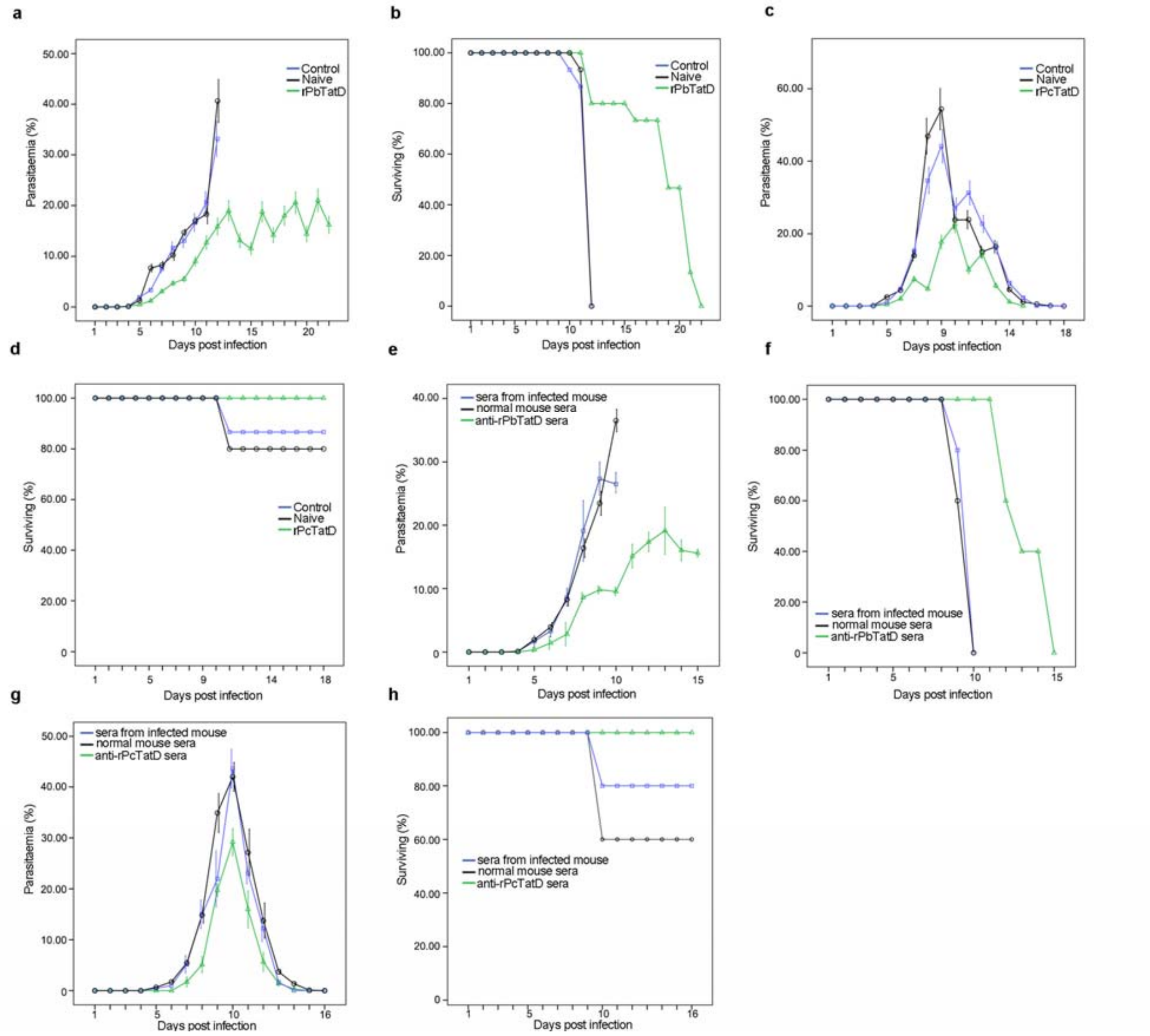

Supplementary Figure 10 | Immunization with PbTatD and PcTatD recombinant proteins is protective in C57BL/6 mice. In (a) and (b), the results of immunization and protection with rPbTatD in C57BL/6 mice are represented. Mice immunized with Freund's adjuvant alone exhibited 1.68-fold higher parasitaemia than the rPbTatD-immunized group on day 12 post-infection; the error bars indicate the standard deviation. The rPbTatD-immunized group survived 10 days longer than the control group before all of the animals died. In (c) and (d), the results of immunization and protection with rPcTatD in C57BL/6 mice are represented. The mice (N=15) immunized with Freund's adjuvant exhibited 3.07-fold higher parasitaemia than the

rPcTatD-immunized group (N=15) on day 8 post-infection; the error bars indicate the standard deviation. In (e) and (f), the results of passive immunization with anti-rPbTatD sera are represented. The group that received serum from a previously infected mouse exhibited 3.30-fold higher parasitaemia than the group that received anti-rPbTatD on day 10 post-infection. The group that received anti-rPbTatD serum survived 5 days longer than the other groups. The error bars indicate the standard deviation. In (g) and (h), the results of passive immunization with anti-rPcTatD sera are represented. The group that received serum from a previously infected mouse exhibited 2.61-fold higher parasitaemia than the group that received rPcTatD-specific serum on day 10 post-infection. The error bars indicate the standard deviation.

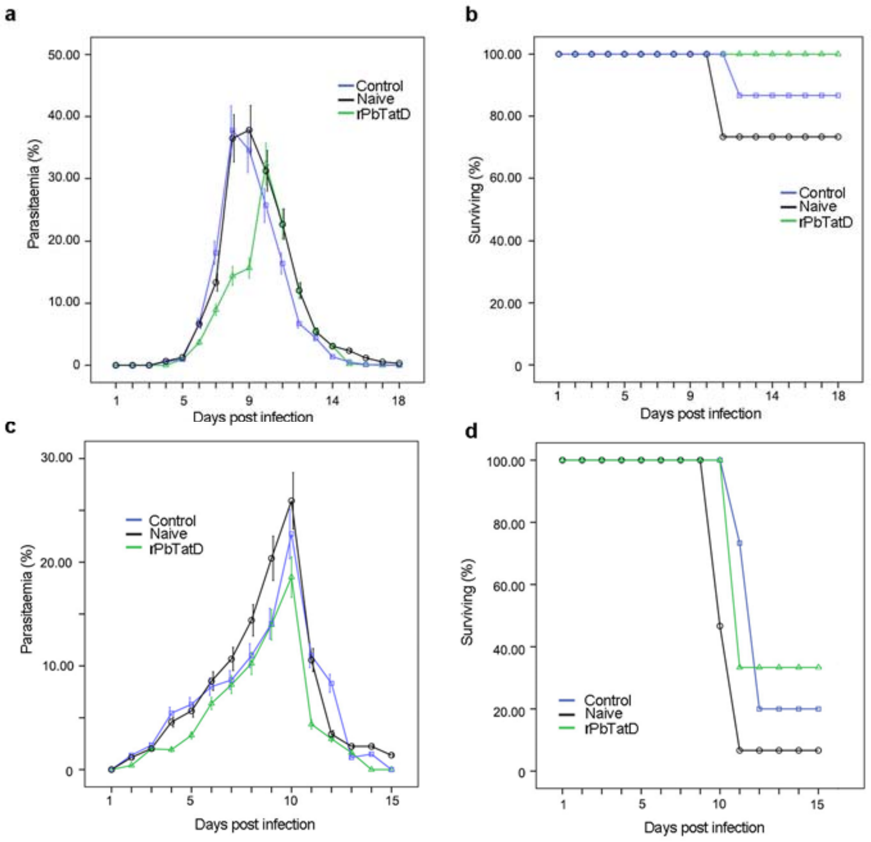

Supplementary Figure 11 | Cross species protection tests. In (a) and (b), the parasitaemia and survival rates of the rPbTatD-immunized mice challenged with *P. chabaudi* AS are shown. The mice immunized with Freund's adjuvant alone and the naïve group exhibited 1.68-fold higher parasitaemia than the rPbTatD-immunized group. All of the mice that survived were immunized with rPbTatD-HIS after challenge. The error bars indicate the standard deviation. In (c) and (d), parasitaemia and the survival rate of rPbTatD-immunized mice challenged with *P. yoelii* 17XL. The mice that were immunized with Freund's adjuvant alone and the naïve group exhibited 1.54-fold higher parasitaemia than the rPbTatD-immunized group. The error bars indicate the standard deviation.

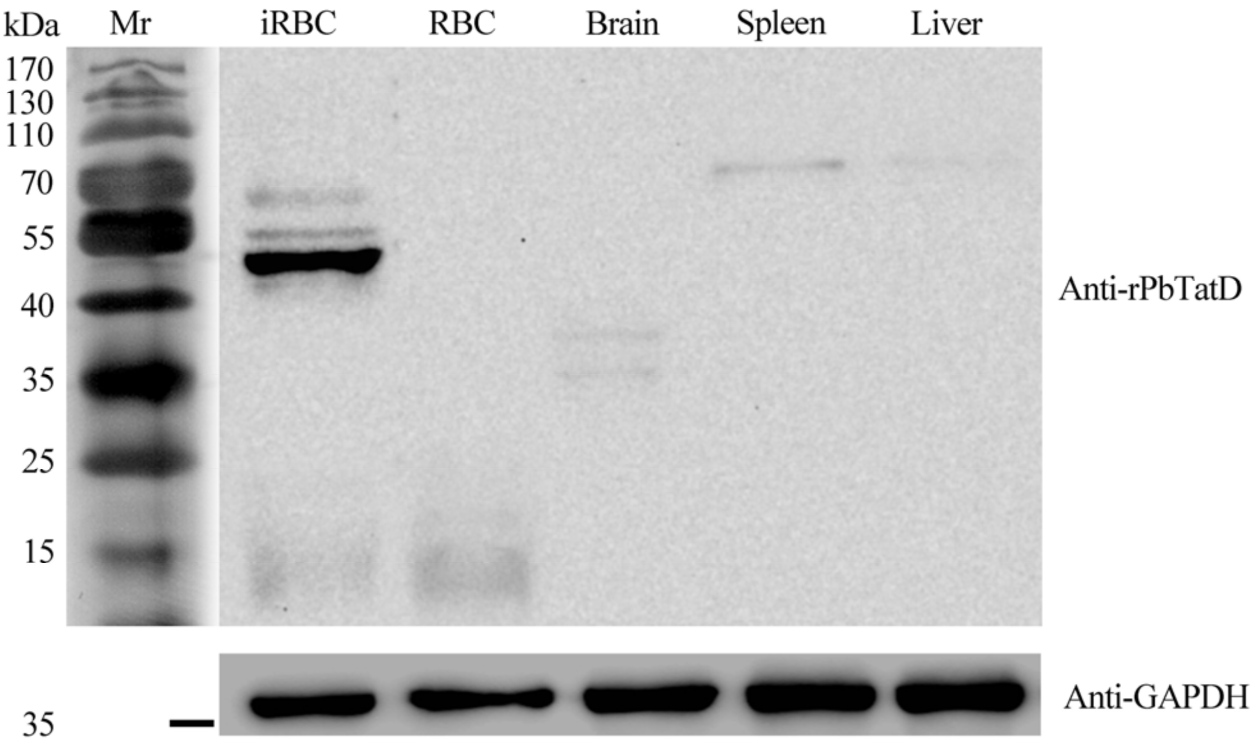

Supplementary Figure 12 | Analysis of the cross-reactivity of anti-plasmodial TatD antibody with host proteins. Mouse tissue proteins were prepared by grinding them in liquid nitrogen, and GAPDH was used as a control. The results showed that no reactivity appeared at approximately 28.9 kDa. The antibody showed weak reactivity with several host proteins in the spleen that were of a greater molecular weight than 28.9 kDa.

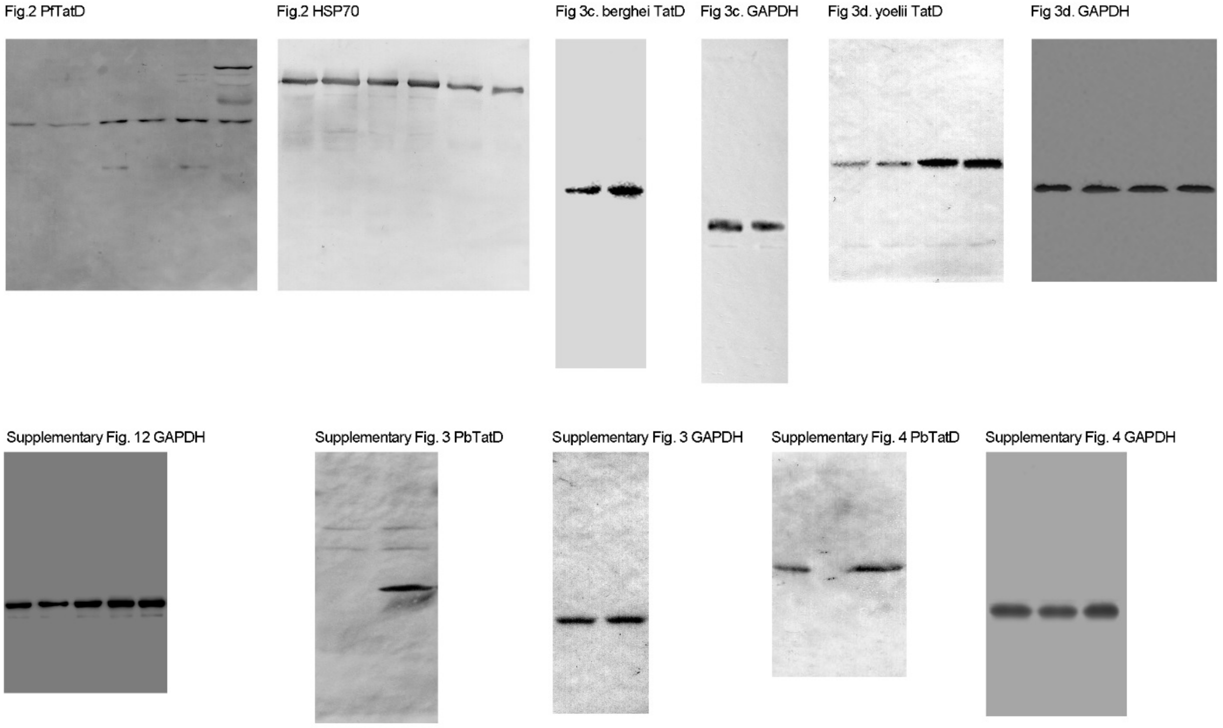

Supplementary Figure 13 | Full size Western blot images. Cropped blots are shown.

<table>
<tr><th>Name</th><th>gene ID</th></tr>
<tr><td>DNase II Oryctolagus cuniculus</td><td>>gi|655901248|ref|XP_008251783.1|</td></tr>
<tr><td>DNase II Galeopterus variegatus</td><td>>gi|667253556|ref|XP_008564879.1|</td></tr>
<tr><td>DNase II Eptesicus fuscus</td><td>>gi|641731686|ref|XP_008156154.1|</td></tr>
<tr><td>DNase II Bos taurus</td><td>>gi|2921841|gb|AAC77367.1|</td></tr>
<tr><td>DNase II Sus scrofa</td><td>>gi|47523470|ref|NP_999361.1|</td></tr>
<tr><td>DNase II Mus musculus</td><td>>gi|35193201|gb|AAH58609.1|</td></tr>
<tr><td>DNase II Homo sapiens</td><td>>gi|3309155|gb|AAC39852.1|</td></tr>
<tr><td>DNase II Poecilia reticulata</td><td>>gi|658892259|ref|XP_008429003.1|</td></tr>
<tr><td>DNase II Danaus plexippus</td><td>>gi|357624166|gb|EHJ75045.1|</td></tr>
<tr><td>DNase II Fowlpox virus</td><td>>gi|9634702|ref|NP_038995.1|</td></tr>
<tr><td>DNase II Penguinpox virus</td><td>>gi|659488174|ref|YP_009046036.1|</td></tr>
<tr><td>DNase II Cynoglossus semilaevis</td><td>>gi|657738098|ref|XP_008327064.1|</td></tr>
<tr><td>DNase II Rana catesbeiana</td><td>>gi|226372402|gb|ACO51826.1|</td></tr>
<tr><td>DNase II Erinaceus europaeus</td><td>>gi|617610019|ref|XP_007524792.1|</td></tr>
<tr><td>DNase II Gallus</td><td>>gi|82541810|gb|ABB81884.1|</td></tr>
<tr><td>DNase II Manacus vitellinus</td><td>>gi|679188080|gb|KFW78929.1|</td></tr>
<tr><td>DNase II Pygoscelis adeliae</td><td>>gi|679131534|gb|KFW62212.1|</td></tr>
<tr><td>cell-death-related_nuclease_7_Acyrthosiphon_pisum</td><td>>gi|641666673|ref|XP_008183818.1|</td></tr>
<tr><td>DNase II Drosophila melanogaster</td><td>>gi|21357117|ref|NP_650672.1|</td></tr>
<tr><td>DNase II Brugia malayi(2)</td><td>>gi|671417128|emb|CDP91568.1|</td></tr>
<tr><td>DNase Burkholderia pseudomallei(2)</td><td>>gi|664640963|emb|CDU32025.1|</td></tr>
<tr><td>DNase Burkholderia pseudomallei</td><td>>gi|660629410|gb|KEO71410.1|</td></tr>
<tr><td>DNase II Paramecium tetraurelia</td><td>>gi|124414750|emb|CAK79853.1|</td></tr>
<tr><td>DNase II Opisthorchis_viverrini</td><td>>gi|663051208|gb|KER27207.1|</td></tr>
<tr><td>DNase II Ancylostoma ceylanicum(2)</td><td>>gi|597894479|gb|EYC42622.1|</td></tr>
<tr><td>DNase II Ancylostoma ceylanicum</td><td>>gi|597887163|gb|EYC35770.1|</td></tr>
<tr><td>DNase II Chlorella_variabilis</td><td>>gi|552831648|ref|XP_005848179.1|</td></tr>
<tr><td>DNase II Dictyostelium fasciculatum</td><td>>gi|470250029|ref|XP_004367246.1|</td></tr>
<tr><td>DNase II S.celegans</td><td>>gi|3183463|sp|Q17778.1|</td></tr>
<tr><td>DNase II Echinococcus granulosus</td><td>>gi|674570361|emb|CDS16432.1|</td></tr>
<tr><td>DNase II Brugia malayi</td><td>>gi|671417128|emb|CDP91568.1|</td></tr>
<tr><td>DNase II Trichuris_suis</td><td>>gi|669333184|gb|KFD73347.1|</td></tr>
<tr><td>DNase II Trichinella spiralis(1)</td><td>>gi|27549463|gb|AAO17050.1|</td></tr>
<tr><td>DNase II Trichinella spiralis</td><td>>gi|117581955|gb|ABK41422.1|</td></tr>
<tr><td>DNase II Trichuris trichiura</td><td>>gi|669225513|emb|CDW53164.1|</td></tr>
<tr><td>DNase Helicobacter pylori</td><td>>gi|672701976|gb|KFH28887.1|</td></tr>
<tr><td>TatD Plasmodium vivax</td><td>>gi|156094961|ref|XP_001613516.1|</td></tr>
<tr><td>TatD Plasmodium cynomolgi</td><td>>gi|457867201|ref|XP_004220524.1|</td></tr>
<tr><td>TatD Plasmodium knowlesi</td><td>>gi|221052222|ref|XP_002257687.1|</td></tr>
<tr><td>TatD Plasmodium falciparum</td><td>>gi|124505869|ref|XP_001351048.1|</td></tr>
<tr><td>TatD Plasmodium vinckei</td><td>>gi|669202848|ref|XP_008626420.1|</td></tr>
</table>

<table>
<tr><td>TatD Plasmodium yoelii</td><td>>gi|23486345|gb|EAA20777.1|</td></tr>
<tr><td>TatD Plasmodium berghei</td><td>>gi|68068543|ref|XP_676182.1|</td></tr>
<tr><td>TatD Plasmodium chabaudi</td><td>>gi|675218753|emb|CDR10616.1|</td></tr>
<tr><td>TatD YEAST</td><td>>gi|465514|sp|P34220.1</td></tr>
<tr><td>TatD Leishmania</td><td>>gi|157865774|ref|XP_001681594.1|</td></tr>
<tr><td>TatD Trypanosoma brucei</td><td>>gi|70834070|gb|EAN79572.1|</td></tr>
<tr><td>TatD Danio rerio</td><td>>gi|317108170|ref|NP_001186951.1|</td></tr>
<tr><td>DNAse Mycobacterium tuberculosis</td><td>>gi|646274917|gb|AIB47597.1|</td></tr>
<tr><td>TatD Mycobacterium tuberculosis</td><td>>gi|378544266|emb|CCE36539.1|</td></tr>
<tr><td>DNAse Salmonella enterica</td><td>>gi|523798481|gb|AGQ64638.1|</td></tr>
<tr><td>DNAse Escherichia coli</td><td>>gi|582993327|gb|EWC54014.1|</td></tr>
<tr><td>DNAse Klebsiella pneumoniae</td><td>>gi|513498480|gb|EPF42044.1|</td></tr>
<tr><td>TatD Escherichia coli</td><td>>gi|628079847|gb|AHY73373.1|</td></tr>
<tr><td>TatD Salmonella enterica</td><td>>gi|81546668|sp|Q9L6M2.1</td></tr>
<tr><td>TatD Chlamydia trachomatis</td><td>>gi|347975445|gb|AEP35466.1|</td></tr>
<tr><td>DNase Chlamydia trachomatis</td><td>>gi|478475377|ref|YP_007725046.1|</td></tr>
<tr><td>DNase Staphylococcus aureus</td><td>>gi|302750381|gb|ADL64558.1|</td></tr>
<tr><td>TatD Staphylococcus aureus</td><td>>gi|537377056|gb|AGU54293.1|</td></tr>
</table>

Supplementary Table 1. The genes coding for DNases and the identification numbers used in the analysis.

Experiment	Primer name	Nucleotide sequence
PfTatD qPCR	PfTatD-FW	ATATCGGATCAAATTTAACTGA
	PfTatD-RV	CAATTTCAGCAAGACAAGTACA
	Pfseryl-FW	AAGTAGCAGGTCATCGTGGTT
	Pfseryl-RV	TTCGGCACATTCTTCCATAA
PbTatD & PyTatD qPCR	P.ber-FW	GAAACAGATGCTCCTTGG
	P.ber-RV	TGGCTCGTTCCTATCCT
	P.yoe-FW	AGTTGGAGGAGTTATTCACA
	P.yoe-RV	CACCAAGGAGCATCTGTT
	butulinFW	AGCAGGCCAATGTGGTAATC
	butulinRV	ACCTGCACGAACACTATCCA
PbTatD D352 construct	D3525'-FW	GGGCCCATTTCACCATTTAATAATTTCCGG
	D3525'-RV	ATCGATTGTCCAATACAATAGTATGATACT
PbTatD KO construct	PbKO5'-FW	CAAGGGCCCAACAATATGAATGACTAGAGG
	PbKO5'-RV	ATTTTTATCGATATATAATATACATTTATTTTGTGCCTA
	PbKO3'-FW	GGATCCAATAATGTATGCAAGTTTTTATATT
	PbKO3'-RV	GCGGCCGCTAGTTTATCATTCATGTGTTATAT
PbTatD complement construct	TatD-FW	TTATAAAACATAGGGGGATCCATGAAATTAATTTTTTACTC
	TatD-RV	AGAAAAACGGGATCTTCTAGATGTATATACACAACTATATTTAAA
	ef1a-TatD-FW	GACCATGATTACGCCAAGCTTGAATTCCTGCAGCCCAGCTTAATT
	ef1a-TatD-RV	GGGCTGCAGGAATTCGATATCTGTATATACACAACTATATTTAAA
	hDHFR-FW	TTATAAAACATAGGGGGATCCATGGTTGGTTCGCTAAACTGCATC
	hDHFR-RV	AGAAAAACGGGATCTTCTAGATTAATCATTCTTCTCATATACTT
Intergration-specific PCR analysis	P1-FW	CTGTTCGATTATAATTGTAGAGGG
	P1-RV	TTAATTCATACACAAACATACAAAAA
	P2-FW	CTTTGATCCCGTTTTTCTTACTTA
	P2-RV	ATATATATTGCCTGGTGAATGTTAA
	P3-FW	ATTTCTTTTTTTAACTGCCGG
	P3-RV	TGGCTCGTTCCTATCCT
	Control-FW	TAAATCAAGGTACCGGCGAATCTTATCAACCATTTATACC
	Control-RV	TTGTATTTCATCGATCTCTTGTGATATTAATCCATACACGCC
Integrated complementation-specific PCR analysis	P4-FW	TTTGGATATTTTCATATATG
	P4-RV	TTTCCCAGTCACGACGTTG
	P5-FW	ATGGTTGGTTCGCTAAACTGCATC
	P5-RV	TTAATCATTCTTCTCATATACTT
	P6-FW	AGAGGGGCATCGGCATCA
	P6-RV	TTGAAAGAATGTCATCTC
Replacement-specific PCR analysis	P7-FW	CCATTTTCTAATTTTATAGTATCT
	P7-RV	TTAATTCATACACAAACATACAAAAA

Supplementary Table 2. The primer sequences for gene identification and for the generation of transfection constructs.
